# Classic and Novel Biomarkers as Potential Predictors of Ventricular Arrhythmias and Sudden Cardiac Death

**DOI:** 10.3390/jcm9020578

**Published:** 2020-02-20

**Authors:** Zornitsa Shomanova, Bernhard Ohnewein, Christiane Schernthaner, Killian Höfer, Christian A. Pogoda, Gerrit Frommeyer, Bernhard Wernly, Mathias C. Brandt, Anna-Maria Dieplinger, Holger Reinecke, Uta C. Hoppe, Bernhard Strohmer, Rudin Pistulli, Lukas J. Motloch

**Affiliations:** 1 Department of Cardiology I, Coronary and Peripheral Vascular Disease, Heart Failure, University Hospital Muenster, Albert Schweitzer Campus 1, A1, 48149 Muenster, Germany; zornitsa.shomanova@ukmuenster.de (Z.S.); christian.pogoda@ukmuenster.de (C.A.P.); holger.reinecke@ukmuenster.de (H.R.); rudin.pistulli@ukmuenster.de (R.P.); 2 Clinic II for Internal Medicine, Paracelsus Medical University, Muellner Hauptstrasse 48, 5020 Salzburg, Austria; b.ohnewein@salk.at (B.O.); c.schernthaner@salk.at (C.S.); k.hoefer@salk.at (K.H.); b.wernly@salk.at (B.W.); m.brandt@salk.at (M.C.B.); u.hoppe@salk.at (U.C.H.); b.strohmer@salk.at (B.S.); 3 Department of Cardiology II, Electrophysiology, University Hospital Muenster, Albert-Schweitzer-Campus 1, A1, D-48149 Muenster, Germany; gerrit.frommeyer@ukmuenster.de; 4 Institute for Nursing and Practice, Paracelsus Medical University, Muellner Strubergasse 21, 5020 Salzburg, Austria; anna.dieplinger@pmu.ac.at

**Keywords:** sudden cardiac death, ventricular arrhythmia, ventricular tachycardia, biomarkers, cardiac biomarkers, heart failure

## Abstract

Sudden cardiac death (SCD), most often induced by ventricular arrhythmias, is one of the main reasons for cardiovascular-related mortality. While coronary artery disease remains the leading cause of SCD, other pathologies like cardiomyopathies and, especially in the younger population, genetic disorders, are linked to arrhythmia-related mortality. Despite many efforts to enhance the efficiency of risk-stratification strategies, effective tools for risk assessment are still missing. Biomarkers have a major impact on clinical practice in various cardiac pathologies. While classic biomarkers like brain natriuretic peptide (BNP) and troponins are integrated into daily clinical practice, inflammatory biomarkers may also be helpful for risk assessment. Indeed, several trials investigated their application for the prediction of arrhythmic events indicating promising results. Furthermore, in recent years, active research efforts have brought forward an increasingly large number of “novel and alternative” candidate markers of various pathophysiological origins. Investigations of these promising biological compounds have revealed encouraging results when evaluating the prediction of arrhythmic events. To elucidate this issue, we review current literature dealing with this topic. We highlight the potential of “classic” but also “novel” biomarkers as promising tools for arrhythmia prediction, which in the future might be integrated into clinical practice.

## 1. Introduction

According to the World Health Organization, cardiovascular disease (CVD) is the number one cause of death. Around 18 million people died of cardiac causes in 2016, accounting for over 30% of all mortality worldwide [1]. Sudden cardiac death (SCD), most often induced by ventricular arrhythmias, is one of the main reasons for CVD-related deaths. Coronary artery disease (CAD) remains the leading cause of SCD with up to 80% of all patients suffering from SCD. Cardiomyopathies like dilated cardiomyopathy account for around 15% of the SCD population, while especially in younger populations genetic disorders are overrepresented [2,3]. Consequently, high-risk populations have been identified, one of the most prominent being heart failure with reduced left ventricular ejection fraction (HFrEF) [4].

Even in this high-risk population, which is prone to develop malignant episodes of ventricular arrhythmias with consecutive SCD [5], antiarrhythmic drug therapy often increases or at best has a neutral effect on cardiac-related mortality [6]. With the beginning of the implantable cardiac defibrillator (ICD) era, a new effective tool for prevention of SCD was available. Indeed, the MADIT- and SCDHEFT trials showed high therapeutic primary prevention efficiency in a high-risk population [7,8]. Patients with severe reduced left ventricular function with ischemic but also with non-ischemic etiology presented a reduced overall mortality after ICD implantation. Based on such promising data, ICD therapy for the prevention of SCD is considered a class I indication in patients with severe impaired left ventricular ejection fraction (LVEF < 35%) [9].

Of note, device therapy is designed to convert tachyarrhythmias following their onset. Therefore, it does not cure the arrhythmogenic disorder. On the other hand, inappropriate ICD-mediated shocks can substantially reduce patients’ quality of life by causing a variety of psychopathological disorders [10]. To add insult to injury, device therapy is associated with frequent surgical complications as well as device and lead failures [11]. Consequently, a significant number of patients receive ICD therapy without any benefit, while suffering adverse events. Therefore, improvements in risk stratification for SCD remain one of the main goals in daily clinical practice. Nevertheless, despite many efforts to enhance the efficiency of risk-stratification strategies by application of electrocardiogram (ECG) parameters, genetic testing, measurements of the autonomic nervous system and novel imaging tools like magnetic resonance imaging (MRI), up to date confirmation of severe LVEF reduction seems to be the only efficient tool [12]. However, while the majority of SCD patients present with preserved left ventricular ejection fraction, this strategy shows a low sensitivity in the general population.

Ventricular tachyarrhythmias are caused by different pathophysiological mechanisms including enhanced automaticity, triggered activity and/or reentry [13,14]. The first two are provoked by cellular phenomena. Enhanced automaticity is characterized by an acceleration of the spontaneous firing rate of the action potential. Consequently, increased automaticity of ventricular myocytes can lead to irregular activation patterns of the myocardium. Triggered activity is characterized by calcium-mediated premature action potentials that arise from early or delayed afterdepolarizations. On the other hand, the most common mechanism of cardiac reentry is a multicellular process involving excitation wave fronts that propagate around zones with impaired conduction and refractory tissue.

These pro-arrhythmic effects are caused by electrophysiological remodeling processes with consequent impaired heterogeneity of cardiac ion channel expression and function within the different regions and layers of the heart. Furthermore, fibrotic processes influence the electrophysiological characteristics of the cardiomyocyte and have a major impact on cardiac conduction [13,14]. All these processes are presented in major cardiac pathologies with increased risk of ventricular arrhythmias, including heart failure (HF) and cardiac ischemia, as well as inherited arrhythmogenic disorders like hypertrophic cardiomyopathy (HCM), arrhythmogenic right ventricular dysplasia (ARVD) or Brugada syndrome. Of note, these mechanisms are often modulated or/and induced by different processes like myocardial necrosis, inflammation, myocardial stress or neurohormonal activation with the involvement of various biological signal proteins. While these proteins are often released during signaling processes, their levels can be measured in patient serum as indicator of signaling activation. Consequently, they can be useful for characterization of normal or pathogenic processes of the heart including electrophysiological remodeling. Indeed, biomarkers have become a useful tool, which refers to a broad subcategory of quantifiable and reproducible characteristics of biological signs. Therefore, they can and should be used for cardiac risk stratification.

Indeed, since the incorporation of aspartate transaminase in the diagnosis of acute myocardial infarction (MI) in the late 1950s, the predictive value of cardiac biomarkers has been an important field of ongoing research. Consequently, “classic” cardiac biomarkers like BNP or troponin, but also inflammatory biomarkers like C-reactive protein (CRP) or high-sensitive (hsCRP), have improved general diagnostic efficiency in various cardiovascular diseases like CAD or HF [15]. This leads to their broad clinical implication in the cardiovascular field. In addition, emerging biomarkers and further on the horizon of categories like myocardial necrosis, inflammation, plaque instability, platelet activation, myocardial stress and neurohormonal activation were investigated in recent years. Indeed, “novel” cardiac biomarkers, like the soluble suppression of tumorigenicity 2 (sST2) protein, have been uncovered as additional tools to improve the management of cardiac disease [16].

While focusing on cardiac arrhythmias, various studies have already explored the implication of the “classic” biomarkers in risk stratification of ventricular arrhythmias and SCD indicating promising results. Furthermore, recent trials have also focused on the potential of the application of “novel” cardiac biomarkers in this clinically important field. However, to the best of our knowledge, research results were not reviewed, yet.

Therefore, in this review we will summarize how biomarkers of cardiac and non-cardiac origin might help predict the risk of ventricular cardiac arrhythmias in different risk populations. Furthermore, besides the potential clinical implication of the “classic” cardiac biomarkers, we review recent results, which investigated the implication of “novel” cardiac biomarkers as potential predictors of fatal ventricular arrhythmias. Of note, further investigations in this exciting field might be translated into novel risk assessment approaches in the future.

## 2. ‘Classic’ Inflammatory Biomarkers as Potential Predictors of Ventricular Arrhythmias

Inflammation is known to play a pivotal role in the pathophysiology of atherosclerosis with consequent CVD. Consequently, “classic” inflammatory biomarkers like CRP and interleukins have been already evaluated in the setting of coronary heart disease [17]. Indeed, chronic inflammation and thrombosis can transform a stable atherosclerotic plaque to an unstable lesion [17]. While CAD is one of the main risk factors for SCD, an association between classic markers of inflammation and malignant ventricular arrhythmias was already investigated in various clinical trials [18]. This topic will be discussed in the following chapter.

### 2.1. C-Reactive Protein (CRP) and High-Sensitive (hs) CRP

Initially discovered as a pathogenic factor, CRP has been known in medicine since 1930. Nowadays CRP is understood to be an inflammatory acute-phase protein, of which levels increase in case of injury or infection. In humans, it is mainly produced in the liver following increased levels of interleukin 6 (IL-6). Furthermore, it is also released by smooth muscle cells of the aorta as well as by fat tissue [19]. The role of CRP in the development of atherosclerotic plaques is well established [20]. CRP stimulates the absorption of low-density lipoprotein (LDL) in the macrophages of endothelial cells, thus contributing to the progression of atherosclerotic plaques and their conversion from stable to unstable condition. This can cause coronary plaque rupture with following ventricular arrhythmias (ventricular tachycardia/ventricular fibrillation (VT/VF)) with consequent SCD [21]. Inflammation, on the other hand, induces structural remodeling of the heart, which promotes an arrhythmogenic substrate [22,23].

Furthermore, inflammation plays an important role in the pathology of ischemic heart disease and HF. Therefore, it is easy to speculate a link between inflammatory markers and CAD or/and HF related arrhythmias. Consequently, previous investigations focused on the association between CRP and/or high-sensitive CRP (CRP assessed by a more sensitive assay to estimate the risk of CAD) and ventricular arrhythmias related to ischemic heart disease or/and ischemic HF. Although the link between inflammation and (ventricular) arrhythmias via ischemic heart disease seems well documented, the question remains whether these inflammatory markers are directly related to the intrinsic pathomechanisms of these malignant events.

The association of inflammatory markers including CRP with MI and SCD has been described in several epidemiological studies. During a follow-up of 13 years of 5888 elderly subjects (aged over 65 years) baseline levels of CRP but also interleukin 6 (IL-6) were linked to the long-term risk of SCD [24]. Similar results were observed in 9758 middle-aged men, when CRP, IL-6 and fibrinogen plasma levels were linked to MI-related death. Nevertheless, in this trial only increased IL-6 levels were found to be an independent risk factor [25]. Further investigations focused on the incidence of ventricular arrhythmias during the acute phase of MI. Hodzic and colleagues observed a positive correlation with increased troponin, but also with CRP [26].

The levels of CRP were also investigated in patients carrying ICDs. Of note, this population at risk has a reliable rhythm monitoring due to the implanted devices. As it turns out, an association between the occurrence of VT/VF with increased serum CRP levels was also described in these patients [27,28].

Furthermore, CRP levels were also investigated in patients with purely non-CAD related arrhythmogenic disorders. A study investigated inflammatory markers in patients with arrhythmogenic right-ventricular dysplasia (ARVD). This inherited rare cardiomyopathy is characterized by scar formation in the right ventricle favoring the incidence of malignant ventricular arrhythmias. When compared to another group of patients with idiopathic right outflow VT, ARVD patients had significantly higher levels of serum CRP. Also, within the ARVD group, Bonny and colleagues observed increased serum levels of CRP 24 h after VT incidence. Interestingly, infiltrates of T lymphocytes were found in myocardial biopsies of ARVD patients, thus suggesting a mechanistic link between inflammatory markers, inflammation and arrhythmias [29]. Other interesting results were described in patients experiencing torsade de pointes (TdP) tachycardia. Of note, this malignant arrhythmia is often promoted by QT-interval prolongation on ECG. Interestingly, CRP elevation corresponded to QT-Interval prolongation. Consequently, the authors speculated that inflammatory cytokines might influence ion channel function with consequent alteration of the QT interval [30].

High-sensitivity C-reactive protein (hsCRP) assays can detect CRP concentrations much lower than conventional CRP assays (down to < 0.04 mg/L). Therefore, they facilitate the detection of low-grade inflammation [31,32]. For this reason, several studies investigating CRP as a potential predictive risk factor for SCD used hsCRP assays for estimation of low-grade inflammatory activity.

The prognostic value of hsCRP for the occurrence of SCD has been evaluated in an epidemiological trial. In healthy men, increased baseline hsCRP levels were associated with a 2.8-fold increased risk of SCD, thus indicating the possibility using this inflammatory marker for identifying high-risk patients [21]. Further trials focused on patients with implanted ICD. Of note, when investigated in patient cohorts following ICD implantation (for primary or secondary prevention), baseline hsCRP levels in patients with appropriate ICD therapy were significantly higher compared to those without ICD therapy. This relationship consisted during the follow-up of 24 months. A baseline hsCRP >3 mg/L was independently associated with appropriate ICD therapy. In contrast, baseline levels of brain natriuretic peptide (BNP) did not show such association, although an increase of BNP during follow-up was significantly associated with appropriate ICD therapy [33]. Blangy and colleagues reported increased levels of BNP and hsCRP in patients experiencing VT amongst 121 ICD patients with history of otherwise stable CAD and a prior history of MI [34]. Furthermore, when investigated in 100 patients with structural heart disease (ischemic or idiopathic dilated cardiomyopathy) who experienced electrical storms compared to those with single episodes of VT/VF or without ICD intervention, higher baseline, hsCRP but also IL-6 and NT-proBNP levels were reported [35].

On the other hand, despite the above evidence regarding hsCRP as a risk predictor of malignant arrhythmias and SCD, some studies did not find such an association. Indeed, in a multicenter prospective observational study performed in 268 patients after MI (>30 days) and LVEF ≤30%, who were indicated for ICD- or cardiac resynchronization therapy-defibrillator (CRT-D)-implantation, no correlation between the occurrence of SCD and/or VT/VF and increased hsCRP was observed (follow-up of two years). Nevertheless, increased hsCRP levels were associated with all-cause mortality, death due to HF and first hospitalization for HF. Therefore, the authors suggested that increased hsCRP levels might predict SCD only in low cardiovascular risk populations [36]. In accordance with this suggestion, Konstantinos et al. found no significant difference in levels of hsCRP, IL-6, tumor necrosis factor alpha (TNF-α) and BNP in stable HF with implanted ICD when comparing patients with ventricular tachyarrhythmia to the arrhythmia free population [37].

A further study investigated hsCRP levels in dialysis patients. These patients commonly have several risk factors for SCD, such as atherosclerosis, left-ventricular hypertrophy with associated fibrosis and endothelial dysfunction. Therefore, they represent a special group with increased risk of SCD. Indeed. Parekhand and colleagues reported higher levels of hsCRP and IL-6 as potential predictors of SCD in this population. Of note, higher levels of these biomarkers were associated with twice the risk of SCD (follow-up of 9.5 years) when compared to patients with lower levels [38].

### 2.2. Interleukin 6 (IL-6)

The cytokine IL-6 is a small signaling protein with inflammatory properties. It is an important mediator of the acute phase response. In atherosclerosis, IL-6 is produced by macrophages in atherosclerotic plaques. Furthermore, it is released by visceral adipose tissue and in the sub-endothelial space. Of note, IL-6 causes an increase of CRP-levels and starts the inflammation cascade [39,40].

As already mentioned above, there is data indicating the predictive role of IL-6 for the occurrence of SCD in epidemiological trials [24,25]. Furthermore, its association with ventricular arrhythmias has been also observed in patients with established CAD. Safranow et al. investigated the interaction between inflammation, metabolic syndrome and arrhythmias in 167 CAD patients. CRP and IL-6 were found to be independent predictors of symptoms of advanced CAD including the incidence of ventricular arrhythmias. The occurrence of metabolic syndrome was strongly related to IL-6. This observation was linked to the contribution of the inflammatory biomarkers in the evolution of insulin resistance, leading to manifestation of metabolic syndrome. Regarding episodes of VT or/and VF, the investigators found a strong association with increased IL-6 and CRP levels. The authors speculated that inflammatory biomarkers could be involved in the transformation of the atherosclerotic plaques into instable lesions, leading to ischemia and respective malignant arrhythmias [40].

As already pointed out above, data on the predictive value of IL-6 in the ICD population is controversial [35,37]. Nevertheless, Streitner and colleagues reported promising results. In 47 patients with implanted ICD (ischemic or dilated cardiomyopathy), significantly higher IL-6 levels were reported at baseline and during follow-up (nine months) in patients experiencing arrhythmic episodes. Indeed, elevated IL-6 serum concentrations were associated with a higher risk of spontaneous VT/VF events [41]. These observations were reassured by the results presented by Cheng and colleagues who investigated a multimarker approach [42]. Nevertheless, this trial will be discussed in the following chapter.

## 3. ‘Classic’ Cardiac Biomarkers as Potential Predictors of Ventricular Arrhythmias

The use of biological markers has been able to improve the accuracy of diagnosis and therapy in cardiovascular patients. In various cardiovascular pathologies, this approach promotes stratification of cardiovascular risk, both during the hospitalization period and the long-term observation period. Indeed, levels of several biomarkers indicate the incidence of malignant cardiovascular events, reflect the dynamics of disease and enhance the efficacy of therapy regimes. “Classic” biomarkers like Troponins are well integrated clinical tools in identifying cardiac damage, but also correlate with the long-term outcome of cardiac patients [43]. The serum level of BNP, a protein secreted by cardiomyocytes during cardiac stress, constitutes a tool already routinely applied in the diagnosis and monitoring of HF patients [44].

Therefore, the role of the described “classic” biomarkers was already extensively investigated in various cardiac pathologies. Interestingly, in the past, several studies dealing with diverse cardiac pathologies associated with ventricular arrhythmias have also focused on their potential role for the prediction of these malignant disorders. These investigations revealed promising results [45]. Consequently, this chapter will focus on the clinical potential of “classic” cardiac biomarkers BNP and NT-proBNP as well as Troponins in dealing with malignant ventricular arrhythmias.

### 3.1. Brain Natriuretic Peptides (BNP and Non-Terminal (NT)-proBNP)

The pre-pro brain natriuretic peptide (pre-proBNP) is a hormone consisting of 134 amino acids, released by ventricular cardiomyocytes during mechanical stress situations like increased volume, stretch and hypertrophy. A part of this protein (consisting of 108 amino acids) splits from the pre-proBNP molecule, resulting in the prohormone BNP (proBNP). ProBNP is further split into two molecules: the biologically active BNP (32 amino acids) and the inactive non-terminal (NT)-proBNP (76 amino acids). NT-proBNP circulates longer in the blood (and thus has a higher concentration) compared to BNP, making it easier to measure in laboratory tests. BNP induces vessel dilation and diuresis, thus reducing preload and afterload, and consequently reducing myocardial stress. It is eliminated by binding to cells expressing BNP-receptors, while NT-proBNP is eliminated through the kidneys. Therefore, patients with renal disease have increased NT-proBNP levels, making its clinical interpretation significantly more difficult. Both BNP and NT-proBNP are established biomarkers of structural heart conditions. Interestingly, their association with ventricular arrhythmias and SCD was also investigated in various trials, while in their elegant meta-analysis Scott and colleagues already highlighted its application in HF patients [46]. Furthermore, novel trials investigated the potential application for risk stratification of ventricular arrhythmogenic disorders when combined with “novel” cardiac biomarkers. However, these studies will be discussed in the next chapter of this review dealing with “novel” biomarker candidates.

Several studies have found an association between increased BNP levels and the occurrence of malignant ventricular arrhythmias or/and SCD [47,48,49]. Furthermore, in their elegant study performed in 521 patients following acute MI, Tapanainen and colleagues elucidated that besides low LVEF, also increased levels of BNP are significant predictors of SCD. Interestingly, the SCD survival curves of patients with and without BNP elevation started to diverge at 20 months after MI, with the split further increasing during the 43 months of follow-up. Consequently, the authors speculated that BNP would indicate ventricular stretch, hypertrophy and fibrosis, which in the long run induce tissue fibrosis and other arrhythmia-related changes of the myocardium. Therefore, BNP could play a role as an indirect predictor of malignant ventricular arrhythmias, as it reflects malignant electrophysiological remodeling processes [50].

Another research group investigated the role of BNP levels in predicting SCD in a high-risk population of 452 patients with HFrEF. During a follow-up of three years, the authors were able to identify BNP as an independent predictor of SCD. In line with Tapanainen and colleagues, they also speculated BNP levels reflect the stage of cardiac remodeling, since the release of this hormone is provoked by similar etiologies, which promote this pathophysiological process (stretch, increased intraventricular pressure etc.) [51]. These results were confirmed by further investigations. Indeed, Watanabe and colleagues found an increased risk of SCD in HFrEF patients when increased BNP levels were combined with left ventricular impairment and dilation parameters as well as non-sustained VTs and diabetes [52]. Furthermore, in this same context, various smaller single-center studies observed potential value of increased BNP levels when used for the prediction of ventricular tachyarrhythmias in the ICD HFrEF population [28,34,53,54,55,56].

There is also evidence that if effective HF therapies can lower BNP, it translates to a better prognosis in terms of malignant ventricular arrhythmias and SCD. Such was the case in the MADIT-CRT study. Effective CRT-D therapy was able to reduce BNP levels after one year. Patients, whose BNP levels were reduced by more than one-third of the baseline value, had a significantly lower risk of subsequent VT/VF or death. The authors suggested that cardiac resynchronization probably led to reverse ventricular remodeling, which in turn reduced the risk of malignant arrhythmias [53].

Based on the promising results described above, it would be tempting to link BNP to specific mechanisms of ventricular arrhythmias, such as the prolongation of the membrane action potential. Of note, in HF this pathology leads to prolonged QTc on ECG with consequent increased risk of VTs and SCD. Vrtovec and his group investigated this exciting topic. In SCD patients, they observed an association between increased levels of BNP and prolonged QTc. Because QT interval is mainly affected by ventricular repolarization, the authors hypothesized that patients with elevated BNP may develop prolonged action potential duration and therefore QT-interval prolongation. They speculated additional BNP induced alterations on a cellular level. Consequently, the authors suggested BNP regulates cardiac calcium metabolism leading to increased calcium entry with resulting electrophysiological abnormalities and ventricular tachyarrhythmias [57].

Further investigations focused on the application of BNP levels in inherited arrhythmogenic disorders such as HCM. This inherited condition is characterized by severe ventricular hypertrophy with or without left outflow obstruction leading to cardiac stress with particularly increased risk of SCD. Consequently, risk stratification in this population is one of the main clinical objectives. Two research groups from Japan investigated the prognostic potential of elevated BNP in HCM patients revealing promising results. Indeed, elevated cTnI but also BNP levels were associated with an increased risk of the incidence of cardiovascular events including VT. Interestingly, combination of both was able to boost the predictive value when compared to a single-marker approach [58]. Minami and colleagues could confirm this observation. The authors observed also a relationship between increased BNP levels and SCD in this population (*n* = 346) indicating BNP as a promising tool for the prediction of malignant arrhythmic events in this inherited arrhythmogenic pathology [59].

Based on its longer half-life and higher concentrations in the peripheral blood (compared to BNP), NT-proBNP is the cardiac marker commonly used to diagnose and control the progression of HF in the daily clinical practice. In accordance with the results described above for BNP and already summarized in the meta-analysis by Scott and colleagues [46], various investigations presented also promising results for NT-proBNP, when applied as predictor of SCD in the HF population.

Elevated intraventricular volume and pressure eventually leads to dilation of the left atrium (LA). Whether such a dilation of LA also has a predictive role for SCD was the research topic of a study group from Spain. In 494 HF patients Bayes-Genis et al. found that, the combination of both increased LA size (>26 mm/m²) and NT-proBNP (>908 ng/L) was associated with an eight-fold increased risk of SCD, resulting in a 25% risk of this event in the follow-up period of 36 months. Consequently, the authors suggested a high specificity of this approach, although the underlying mechanisms for their observations remain unknown [60].

Indeed, higher NT-proBNP levels seem to be associated with increased occurrence of ventricular arrhythmias and/or SCD in patients with HF due to ischemic and non-ischemic etiology [61,62]. However, there is yet insufficient evidence, whether any cardiac biomarker qualifies as a powerful risk predictor for malignant arrhythmias and/or SCD in this population. Nevertheless, prediction of these malignant events in HF patients is one of the main objectives of present translational research. In an ideal clinical scenario, the decision whether a prophylactic ICD implantation is indicated in HF patients, should depend on their assessed risk of malignant arrhythmias and/or SCD. As of today, HF patients undergo prophylactic ICD implantation based on present cardiac societies’ guidelines (HF symptoms combined with a significantly reduced LVEF). Therefore, in order to investigate an additive application of biomarkers in this population, several biomarker studies (including NT-proBNP) tested their potential as predictors of mortality and/or arrhythmias following ICD-implantation, with encouraging results [35,63,64,65,66,67]. Notably, arrhythmic events are easy to monitor in this population, due to the implanted device systems. In one of the larger studies, Cheng and colleagues investigated 1189 patients with HFrEF following ICD implantation for primary prevention of SCD. During a follow-up period of four years, 137 patients had appropriate ICD shocks while 343 patients suffered from death for various reasons. Nevertheless, in this study only higher IL-6 levels were able to predict the occurrence of appropriate ICD shocks while all investigated biomarkers (CRP, IL-6, TNF-α, NT-proBNP and troponin T) presented a higher risk of all-cause mortality. Therefore, based on their results, the investigators suggested a combined biomarker score reflecting all-cause mortality, in order to identify patients who are unlikely to benefit from primary prevention through ICD [42].

Following out-of-hospital resuscitation, measured NT-proBNP show distinguishing properties between underlying ischemic und non-ischemic heart disease, as well as in terms of survival of patients. Aarsetøy and colleagues investigated the application of serum copeptin and hscTnT but also NT-proBNP in the event of SCD. They collected blood samples from 77 patients following out-of-hospital resuscitation due to VF, and observed promising results for NT-proBNP. Of note, the biomarker was significantly higher in patients with heart disease without MI and in non-survivors compared to survivors, which also in this population supports the hypothesis of its predictive value [68].

Because NT-proBNP, in contrast to BNP, undergoes renal elimination, its serum levels are also increased due to renal dysfunction [69]. Therefore, several studies focused on NT-proBNP in patients with kidney disease. An association was found between SCD and elevated NT-proBNP, but also cTnI levels, in hemodialysis patients [70,71,72]. However, different cut-off serum levels were suggested. Winkler et al. concluded serum levels over 9252 pg/mL being predictive of SCD (two-fold increased risk) [72], while Kruzan et al. proposed a cut-off > 7350 pg/mL (three-fold higher risk of SCD) [71]. Of note, this group also observed a higher predictive value for NT-proBNP than for cTnI. The authors speculated that volume overload with consequent ventricular stretching, may drive NT-proBNP elevation. Nevertheless, they did not exclude decreased renal clearance as a potential reason for the NT-proBNP elevation in this patient group [71]. On the other hand, when measuring levels of NT-proBNP during a four-year follow-up in dialysis patients with type 2 diabetes mellitus, Winkler et al. were able to identify subgroups of patients with increased risk of SCD. Of note, patients with higher baseline NT-proBNP, which decreased over 10 percent in the follow-up measurements, had lower adjusted relative risk of SCD than patients with stable levels [72].

Some further research was performed in patients with HCM. Nevertheless, when investigated in 847 HCM patients, NT-proBNP levels were a significant predictor of HF and transplant-related deaths but not for SCD or appropriate ICD shocks [73]. Similar findings were revealed by Rajter-Salwa and his group who investigated the relationship between biomarkers (hs-TnI and NT-proBNP) and the calculated five-year risk score for SCD in 46 HCM patients [9]. Notably, no difference between patients with higher and lower NT-proBNP levels was noted, indicating NT-proBNP to be a poor predictor of ventricular arrhythmogenic events in the HCM population [74].

As previously mentioned, as an established biomarker in management of HF, NT-proBNP seems also to be an additive useful tool, when applied for risk stratification for ventricular arrhythmias or/and SCD in this population. Nevertheless, promising results were also revealed when applied in the “healthy” population. Of note, NT-proBNP levels seem to be associated with increased frequency of ventricular ectopy [75,76]. Furthermore, when investigated in a prospective case-control study in 32,828 healthy nurses (Nurse Health Study), an association between NT-proBNP at baseline and the risk of SCD during 16 years of follow-up was observed. NT-proBNP levels over the cut-off of 389 pg/mL had a five-fold increased risk of SCD, indicating even in the “healthy” population a potential value of this biomarker [77].

### 3.2. Troponins

The troponin complex includes three subunits and is positioned on the thin filaments of the striated muscles. These subunits are troponin T (TnT), troponin I (TnI) and troponin C (TnC). TnT is a protein, which connects the troponin complex with tropomyosin. TnI controls the binding of actin with myosin. The role of TnC is to connect tropomyosin with calcium. While TnC has the same structure in both the skeletal and heart muscle, in the heart TnT and I have different amino acid compositions. Thus, both cardiac troponins (cTnI and cTnT) can be identified in the blood as specific biomarkers of the heart. Of note, assays for high-sensitivity (hs) Troponins provide a more sensitive measurement allowing the detection of lower concentrations [78]. Therefore, they are implicated in daily clinical practice.

Cardiac troponins (cTnT and cTnI) are biomarkers of myocardial injury mostly released during necrotic processes often caused by myocardial ischemia. Necrosis promotes the replacement of cardiac myocytes with fibrotic tissue as well as further electrophysiological remodeling which predispose ventricular arrhythmias with eventual SCD. However, necrosis is not the only cause for Troponin release. A small free pool of TnT is situated in the cytosol. Therefore, prolonged leakage might be observed during the degeneration of myofilaments in irreversibly injured cells [79]. Besides being markers of cardiac damage, several studies investigated the application of levels of Troponins as potential tools in the risk assessment of malignant arrhythmias.

Indeed, as we describe in the previous chapters of this review, several trials showed promising results when evaluating troponins in terms of the occurrence of ventricular arrhythmias or/and SCD [28,42]. Liu et al. investigated the possible association between levels of Troponin and ventricular arrhythmias in 218 patients with chronic HF. In the setting of severe decompensated HF, patients with positive cTnI (>0.5 ng/mL) were more likely to develop ventricular arrhythmias than patients with negative troponin. The authors speculated that patients with decompensated HF might suffer from minimal myocardial injury or “microinfarction” causing sub endocardial ischemia or increasing wall stress with consequent myocardial necrosis [80].

As already mentioned above, high-sensitivity troponin assays enable the detection of lower troponin levels. Therefore, they facilitate earlier diagnosis of MI or/and other cardiac stress situation. Their application for prediction of malignant arrhythmogenic events was also investigated in several trials.

A larger longitudinal study from the USA evaluated the association between the levels of hsTnT and SCD in 3089 older subjects (ambulatory participants in the Cardiovascular Health Study) during a follow-up of 13 years. Indeed, even after adjustment for typical risk factors, elevated baseline hsTnT levels were associated with the incidence of SCD. The authors speculated hsTnT to reflect cardiomyocyte injury caused by possible ageing processes or unrecognized coronary disease with consequent scar formations as potential substrate for the incidence of ventricular arrhythmias [81].

Of note, this hypothesis is in line with studies performed in CAD patients. When investigated with other biomarkers (hsCRP, sST2, BNP) in 1946 CAD patients with preserved left-ventricular function (mean follow-up of 76 ± 20 months), elevated sST2 but also hsTnT (≥15 ng/mL) were the strongest predictors of SCD (followed by hsCRP and BNP) [49].

Interestingly, besides ischemic heart disease, hs-troponins may also be of predictive value when dealing with other cardiomyopathies. Indeed, patients with non-ischemic cardiomyopathy and increased hsTnT levels may have increased risk of SCD, as well. Two investigator groups investigated this exciting issue in patients with dilated cardiomyopathy. They compared hsTnT to conventional TnT, in terms of predicting cardiovascular events including SCD. Both groups found hsTnT to be a better independent predictor than TnT in multivariate analyses [82,83]

Furthermore, Kubo and colleagues investigated hsTnT as a potential marker for prediction of adverse events in 183 HCM patients. They found that elevated hsTnT, but also the degree of elevation, were associated with a higher risk of adverse cardiovascular events including the incidence of sustained VT. The authors supposed that increased hsTnT in HCM patients may reflect relative myocardial ischemia promoted by an imbalance between the hypertrophy of the myocardium and insufficient coronary arterial supply [84].

## 4. ‘Novel and Alternative’ Biomarkers as Potential Predictors of Ventricular Arrhythmias

As already mentioned above, cardiac biomarkers are protein components of cell structures that are released into the blood stream when myocardial injury occurs. Consequently, they have a major impact on the diagnosis, risk stratification, and treatment of patients with various cardiac pathologies and symptoms including chest pain with suspected acute coronary syndrome or during evaluation of acute exacerbations of HF. In recent years, active research efforts have brought forward an increasingly large number of “novel and alternative” candidate markers candidates of various pathophysiological origins. Investigations of these promising biological compounds have revealed exiting and encouraging results when dealing with cardiovascular pathologies. Interestingly, their diagnostic, prognostic and/or therapeutic utility was already investigated in the first clinical trials evaluating ventricular arrhythmogenic disorders. While trials with “classic” biomarkers are summarized in Table 1, Table 2 and Table 3, new promising biomarkers candidates are presented in Table 4, Table 5 and Table 6 and will be discussed in the following chapter.

### 4.1. Soluble ST2 (sST2)

ST2 is a member of the interleukin 1 receptor family. Originally, this protein was linked to myocardial dysfunction, fibrosis, and remodeling [100]. Interestingly, upregulation of soluble ST2 (sST2) was also shown to be related to mechanical stress of the heart with consequent cardiac damage. Of note, there are two known isoforms, which both are associated with cardiac pathologies. While sSt2 is soluble, the second isoform St2 is a receptor, bound to the cell membrane [101]. Their role in cardiac pathophysiological processes involving progression of coronary atherosclerosis, but also cardiac remodeling with consequent fibrosis, has been uncovered in recent years [100]. Of note, their function was shown to depend on interleukin 33 (Il-33). Il-33 binds to the ST2 receptor in order to reduce cardiac damage during cardiac stress. Nevertheless, tethered with sST2, Il-33 is unable to become involved into further cellular pathways, resulting in the potential loss of cardioprotective characteristics [102,103]. Consequently, higher levels of sST2 are linked to more severe stress responses in the heart [103]. On the other hand, sST2 seems to be involved in the pathophysiology of ischemic events. Of note, serum levels are associated with ischemic damage and remain high, even in the post-MI period. Since recovery of left ventricular function is impaired in patients with higher sST2 levels, sST2 is speculated to play an important role in remodeling following an acute ischemic event [104]. Logically, further efforts were made to integrate sST2 into daily clinical practice of dealing with cardiovascular patients.

Indeed, higher sST2 levels (above 36.3 ng/mL) are associated with adverse outcomes in patients with HF [105]. Since NT-proBNP and sST2 are both elevated in this pathology and are part of two different pathological pathways, combining them as part of a risk assessment strategy was the next logical step. In fact, among symptomatic HF patients, sST2 concentrations are strongly predictive of mortality and might be useful in risk stratification when used alone or together with NT-proBNP [106]. Consequently, a moderate benefit in the risk assessment of HF patients was made when measurements of sST2 were combined with NT-proBNP. These results, led to the proposal of a “solid” threshold of sST2 levels in HF patients [106,107].

Since the HF population is known to be at high risk of ventricular arrhythmias with consequent SCD, the application of this strategy was also investigated for the prediction of these malignant events. In their elegant case-control study, by analyzing data from the MUSIC registry (three-year multicenter registry of ambulatory HF patients with New York Heart Association functional class (NYHA) II-III, and LVEF ≤ 45%, Pascual-Dual and colleagues were able to demonstrate that higher sST2 levels are associated with SCD. Indeed, 34% of patients with sST2 levels above 0.15 ng/mL developed SCD while 74% of patients with both increased sST2 and NT-proBNP levels experienced this fatal event. Therefore, the authors postulated that this combination might be a valuable clinical tool for predicting SCD in HF patients [91]. Nevertheless, these enthusiastic results could not be fully reproduced by further trials. In a subgroup analysis of the HF-ACTION trial, adding novel biomarkers such as sST2 and galectin 3 to NT-proBNP levels in the risk calculation model, showed a strong association with death by pump failure. Yet, there was only a weak improvement while assessing for SCD [89].

However, in patients with mildly symptomatic HF evaluated during the MADIT-CRT trial, a 10% elevation of sST2 levels alone over one year, was shown to be predictive of increased risk of onset of ventricular arrhythmias and death. Nevertheless, in the same study it was shown, that an elevated sST2 baseline is not directly predictive of ventricular arrhythmias [90].

Further, investigations were performed in patients treated with ICD for primary prevention. Since the ST2 protein is a marker of myocardial stress, sympathetic hyperactivation and neuro-hormonal axis dysfunction [28], one might speculate that in the ICD population with HF, sSt2 levels could reflect alterations of the electrophysiological substrate and thus identify patients at a higher risk of shock therapy. Of note, these pathophysiological alterations are more common in patients suffering from metabolic syndrome. Therefore, in their elegant study Sardu and colleagues focused on this specific population at risk. Interestingly, in these patients, sST2 values could differentiate patients with a higher risk of ICD therapy, and worse prognosis [28].

Other studies focused on further specific risk populations. Mitral annulus disjunction is a displacement of the mitral valve. Since it is accompanied by mitral annular myocardial fibrosis, it is a mechanism proposed for the development of ventricular arrhythmias with potential consequent SCD [108]. In this population, patients suffering from ventricular arrhythmias had higher circulating levels of sST2. Indeed, while combined with LVEF and fibrosis assessed by late gadolinium enhancement on MRI, sST2 measurements were able to improve risk stratification in this specific risk population [109].

### 4.2. Galectin-3

Galectins are a family of proteins defined by two characteristics: functionally a beta-galactoside affinity and structurally a conserved carbohydrate recognition domain (CRD). Initially, these proteins were only thought to play a significant role in embryogenic processes. However, further research uncovered, galectins are important players in various physiological and pathophysiological processes including immune activation [110].

The galectin family members are expressed in three different structural forms: dimeric, tandem or chimera. Of all discovered chimeric structural forms, galectin-3 is the only protein with a N-terminal protein-binding domain and a C-terminal carbohydrate-recognition domain. The protein is expressed in various tissues including lung, kidney, as well as the heart. Consistent with other members of the lectin family, this soluble beta-galactoside-binding protein is activated as a response to tissue damage [111]. Galectin-3 is active on both the intracellular and/or the extracellular levels. On the cellular level, it regulates messenger ribonucleic acid (mRNA) splicing and contributes to the regulation of anti-apoptotic signaling [112], while extracellularly it is secreted by macrophages and is involved in the recognition of pathogens as well as in acute chronic inflammation processes [113,114]. Furthermore, this protein seems to be a potent mitogen for fibroblasts [115]. Therefore, galactin-3 represents an intriguing link between inflammatory and fibrotic processes, which are frequent findings in various cardiac pathophysiologies, including HF [116].

Indeed, when measured in the general population, elevated levels of galectin-3 are associated with higher incidence of CVD, but also with an elevated risk of all-cause mortality [117]. Especially in recent years, this protein was shown to be a useful complementary biomarker in prognosis and risk stratification of HF patients [118]. However, as already mentioned above, concerning prediction of SCD in this population at risk, first results adding novel biomarkers including galectin-3 to NT-proBNP for risk assessment, showed only weak improvement while assessing for this malignant event [89]. On the other hand, together with osteopontin, Francia and colleagues evaluated a possible association of galectin-3 levels with the incidence of sustained VT/VF in 75 newly implanted ICD-HF-patients. Of note, even after correction for other risk factors, during a follow-up of over two years, plasma levels of galectin-3 predicted sustained VT/VF in HF patients at high risk of SCD [88].

In various arrhythmogenic pathologies including genetic disorders, tissue inflammation and fibrosis are key processes of electrophysiological remodeling. Therefore, one might speculate that there will be further clinical applications of galectin-3 as a potential tool for prediction of ventricular arrhythmias. Consequently, further studies focused on genetic disorders like ARVD and HCM. Of note, both pathologies are mainly characterized by defective genes responsible for connective tissue structure, resulting in remodeling including tissue inflammation and fibrosis with consequent ventricular arrhythmias [92]. Indeed, in a small study (conducted in 24 patients with ARVD vs. 29 control patients) galectin-3 levels were shown to be increased in patients with ARVD. Furthermore, they were predictive for the onset of VT as well as VF. Therefore, the authors postulated, galectin-3 as a potential biomarker involved in the onset of ARVD. A further study investigated a possible association with risk prediction of SCD in HCM. Of note, in this population, five-year risk of SCD is routinely assessed using a standard questionnaire outlined in the 2014 European Society of Cardiology guidelines [9]. The authors observed a positive correlation between the estimated five-year risk of SCD and serum levels of galectin-3, thus indicating an additive tool for SCD-prediction in this population [95].

### 4.3. Heart-Type Fatty Acid Binding Protein (H-FABP)

Heart fatty acid-binding protein (H-FABP) is ubiquitous in myocardial cells. Therefore, upon myocardial membrane injury H-FABP is released in the bloodstream [119]. Of note, peak levels are observed three hours following an MI [120]. Consequently, H-FABP was established as a marker of ongoing myocardial membrane damage and has been reported to be a useful indicator for future cardiovascular events [121]. Therefore, further trials explored its potential application in predicting arrhythmogenic events in high-risk populations. In 107 consecutive patients with cardiomyopathy, who had received an ICD, circulating serum H-FABP levels >4.3 ng/mL, but not Troponin T levels, were a significant independent prognostic factor for the incidence of appropriate shock therapy or/and cardiac death. Furthermore, assessment of subgroups showed that H-FABP levels could be used to anticipate event-free periods in patients with ICD and additive amiodarone therapy. Indeed, the outcome of patients receiving ICD for primary as well as secondary prevention was predictable via H-FABP levels [85].

A further study investigated the application of myocardial membrane injury assessed by H-FABP levels in Brugada syndrome. Of note, this genetic disorder is defined by inherited sodium channel dysfunction with consequent risk of SCD [122]. Also in this high risk population serum H-FABP levels (>2.4 ng/mL), but not Troponin T levels, were an independent prognostic factor for appropriate ICD shocks due to VF (during a five-year follow up) indicating H-FABP as a promising biomarker for the prediction of malignant ventricular arrhythmias [93].

### 4.4. Metalloproteinases (MMP) and Procollagens

Metalloproteinases (MMPs) are enzymes mainly concerned with the turnover of extracellular matrix. Their role in the development of post-infarction scar tissue is a growing field of investigation. Indeed, these proteins are key enzymes involved in post-MI remodeling, including processing of cytokines and extracellular matrix (ECM) substrates to regulate the inflammatory and fibrotic components of myocardial wound healing. Furthermore, these enzymes are upstream initiators with regulatory functions in cell signaling cascades [123]. Consequently, in HF patients, levels of diverse MMPs seem to reflect the progression of cardiac remodeling [124]. Therefore, as reflectors of cardiac turnover processes with consequent remodeling, they might be suspected as useful predictors of ventricular arrhythmias. Indeed, in 74 HF patients with implanted ICD, the ratio of MMP-9 and the tissue inhibitor of matrix metalloproteinase 1 was able to predict tachyarrhythmic events necessitating appropriate interventions, indicating further potential future applications in this clinical field [87].

While cardiac remodeling is one the main pathophysiological characteristics of hypertrophic cardiomyopathy, a further study focused on this genetic disorder. Indeed, in a population of adolescent HCM patients, MMP-3 levels were significantly higher in patients prone to ventricular arrhythmias. However, when adjusted for age, the effect was attenuated, indicating the need for further research in this exciting field [94].

Previous investigations already focused on other biomarkers, which were known indicators of excessive turnover of the extracellular mass of the heart. They included circulating procollagens. While these compounds were linked to worsening of HF and the function of the left ventricle, further studies explored possible associations with the incidence of ventricular arrhythmias [125]. Indeed, in ICD patients implanted for spontaneous sustained VT due to ischemic heart disease, incidence of VT could be linked to high type I aminoterminal peptide (PINP) and low procollagen type III aminoterminal peptide (PIIINP) levels. Nevertheless, these markers presented a low specificity.

### 4.5. Endothelin

As one of the most potent vasoconstrictive peptides, the endothelium-derived factor endothelin became a novel objective of research in the late 1980s [126]. Endothelin 1 (ET 1) not only leads to the stimulation of interleukin expression in monocytes and increases platelet aggregation, but also stimulates expression of growth factors. EtA and EtB are the predominant receptors activated via endothelin. EtA is exclusively expressed on vascular smooth muscle cells and has a greater selectivity for ET1 [127]. Furthermore, endothelin seems to be a contributing factor in the development of chronic hypertension [128]. Nevertheless, in contrast to other myocardial biomarkers, endothelin 1 has very early been the matter of investigation in the pathophysiology of cardiac arrhythmias. Indeed, in animal models, endothelin is associated with the incidence of ventricular arrhythmias [129,130] but also with ECG modulation including QTc prolongation [130]. In addition, endothelin was linked to ischemia induced ventricular arrhythmias [131] and arrhythmogenic responses during myocardial reperfusion [132]. Several mechanisms are proposed to be activated via ET1 to promote arrhythmic events. Nevertheless, early afterdepolarizations triggered by ion channel remodeling, but also sympathetic activation, were suggested to be the main causes of ET1 induced arrhythmias [133,134,135]. Inspired by these promising results, further research focused on patients with decompensated HF. Of note, this population is characterized by increased neurohumoral activation with a higher rate of ventricular arrhythmias and SCD [4,128,129]. Et1 levels, as well as renin-angiotensin-aldosterone-system (RAAS) activity, but also interleukin 6 and TNF-α were assessed in 83 of those patients. Indeed, 24 h Holter-monitoring revealed an association of Et1 levels and ventricular ectopy [136].

A further study explored the application of ET1 measurement in ICD-patients (implanted for multiple underlying conditions). ET1 levels were significantly increased one hour and even one minute after shock therapy, giving further evidence, that the potential biomarker plays an important role in the development of malignant arrhythmogenic events [137]. Nevertheless, despite promising findings in basic research studies as well as in the first clinical trials, the role of ET1 as potential predictor of ventricular arrhythmias still needs to be evaluated.

### 4.6. Uric Acid

Uric acid is the final product of the purine metabolism. In recent years, serum uric acid has gained interest as a determinant of cardiovascular risk. Indeed, patients with hyperuricemia are at higher risk of cardiovascular events [138]. Furthermore, high serum levels are a strong, independent marker of poor prognosis in HF [139]. Consequently, they also seem to be associated with the incidence of ventricular arrhythmias in this high-risk population. Indeed, in a smaller trial in 56 HF patients with implanted ICD for primary prevention, higher uric acid levels were linked to the development of ventricular tachyarrhythmias [86]. Similar results were revealed in patients with diagnosed left-ventricular hypertrophy. In this population uric acid levels were shown to be an independent predictor of the occurrence of VT during Holter-monitoring [140].

### 4.7. Other Promising Biomarkers

Besides the already discussed promising biomarkers, several trials investigated further biomarkers of various origin. Therefore, we would also like to provide a brief overview of this growing topic of ongoing investigation.

Fibrinogen is a glycoprotein involved in clotting processes. Furthermore, it is a known promotor of revascularization and wound healing, but also acts as an acute-phase protein, which is secreted in response to systemic inflammation and tissue injury [141]. Consequently, fibrinogen plasma levels were shown to be higher in patients suffering from CVD, as indicated by a subgroup analysis of the Framingham population [142]. Nevertheless, data available from the PRIME study (multicenter prospective cohort designed to identify risk factors for coronary heart disease) could not reveal an association with SCD when assessed with other biomarkers such as IL-6 or CRP [25]. Differing results were presented by Kunutsor and colleagues [96]. Interestingly, when investigated in a bigger cohort including 1773 middle-aged men who were followed up for 22 years, fibrinogen levels were positively associated with the risk of SCD. However, addition of plasma fibrinogen to a SCD risk prediction model containing established risk factors was not able to improve risk discrimination in this population [96]

Impaired kidney function is a known cardiovascular risk factor. As already mentioned, this population is also at higher risk of SCD [143]. In their elegant trial, Deo and colleagues investigated a possible association between SCD and established biomarkers of renal function in an elderly population without prevalent CVD at baseline. During a follow-up of more than 10 years, the authors were able to uncover that impaired kidney function assessed by cystatin C, but not by creatinine levels or glomerular filtration rate, are linked to SCD events in the future [144].

Osteopontin is an extracellular structural protein. As an organic component of the bone, it is involved in bone-remodeling processes [145]. Furthermore, while it is expressed in a range of immune cells, it is also involved in immunity [146]. One study focused on the possible connection between osteoponin levels and the incidence of ventricular arrhythmias. As already mentioned above, Francia and colleagues investigated levels of osteoponin and galectin-3 in HF patients with implanted ICD. Indeed, higher plasma levels were predictive of the incidence of sustained VT/VF, indicating this potential biomarker as a clinical promising tool requiring further investigation [88].

Growth differentiation factor-15 (GDF-15) is a stress-responsive transforming growth factor-ß-related cytokine. It increases and is independently related to an adverse prognosis in systolic, but also diastolic HF [147]. Furthermore, it was also suggested to be a prognostic biomarker in the evaluation of short- and long-term outcomes in ST-elevation myocardial infarction (STEMI) patients [148]. Interestingly, in STEMI patients with VF complications, levels of GDF 15 seem to be increased and are also predictive when assessing short-term mortality [149].

## 5. Summary

Recent studies have identified the significance of serum biomarkers as risk factors for ventricular tachyarrhythmias. Beyond the established clinical risk factors, elevations of the “classic” biomarkers like BNP and NT-proBNP, as well as troponins were already elucidated as potential predictors of SCD in various populations at risk. Inflammatory biomarkers seem also to be associated with ventricular arrhythmias and may have a significant role in their pathogenesis. Furthermore, recent studies have investigated “novel” biomarkers originating from various pathophysiological contexts, like sST2, galectin-3 or H-FABP. In the HF population at risk in particular, these substances indicate a promising potential for prediction of malignant arrhythmic events. Furthermore, their application might also be useful in inherited arrhythmogenic pathologies. Combining these biomarkers in a multimarker approach might further improve risk assessment strategies. Nevertheless, further translational research is necessary to elucidate the potential of these promising biological compounds in dealing with ventricular arrhythmias and SCD.

## Figures and Tables

**Table 1:** Predictive value of “classic” biomarkers in heart failure.

Heart Failure	Biomarker	Underlying Condition	Pacemaker/ICD	Arrhythmias	Outcome	Number of Patients	Study Design	FU-Duration	Specific Endpoint	Effect on SCD
Biasucci et al., 2006 [27]	CRP	ICM	ICD	VT/VF	CRP is associated with VT/VF	65	Prospective, single center	-	Appropriate ICD shocks for sVT/VF	SCD not directly investigated
Theuns et al., 2012 [33]	hsCRP, BNP	CHF	ICD	VA	Independently associated with ICD appropriate therapy	100	Prospective, single center	24 months	Appropriate ICD therapy, VA	Independent predictor of SCD *
Blangy et al., 2007 [34]	hsCRP, BNP	ICM	ICD	VT	hsCRP and BNP associated with VTs	121	Prospective, single center	1 year	VTs	SCD not directly investigated
Streitner et al., 2009 [35]	hsCRP, IL-6, NT-proBNP	DCM, CAD	ICD	VT/VF	Correlation with occurrence of electrical storm	86	Prospective, single center	9 months	VT/VF or electrical storm	SCD not directly investigated
Biasucci et al., 2012 [36]	hsCRP	ICM	ICD/CRT-D	VT/VF	Not associated with SCD or VT/VF	268	Prospective, multicenter (CAMI-GUIDE study)	2 years	VT/VF or SCD	No effect
Kontantino et al., 2007 [37]	IL-6, TNFα, hsCRP, BNP	CHF	ICD	VT/VF	No correlation with VT/VF	50	Prospective, single center	152±44 days	VT/VF	SCD not directly investigated
Streitner et al., 2007 [41]	IL-6	ICM	ICD	VT/VF	Associated with VT/VF	47	Prospective, single center	9 months	VT/VF	SCD not directly investigated
Cheng et al., 2014 [42]	IL-6, CRP, TNFα-receptor II, pro-BNP	CHF	ICD	VA	IL-6 predictive for appropriate ICD shocks	1189	Prospective, multicenter (PROSe-ICD study)	4 years	Appropriate ICD shock	IL-6 independent predictor of SCD *
Berger et al., 2002 [51]	BNP	CHF	None	SCD	Independent predictor of SCD	452	Prospective, single center	3 years	SCD	Independent predictor of SCD
Watanabe et al., 2006 [52]	BNP	CHF	None	SCD	Associated with SCD when combined with echo parameters, nsVTs and diabetes	680	Prospective, multicenter (CHART study)	-	SCD	Factor associated with SCD
Medina et al., 2016 [53]	BNP	CHF	ICD/CRT-D	VT/VF	Independent predictor of VT/VF	1197	Sub-study, prospective, multicenter (MADIT-CRT study)	1 year	VT/VF	SCD not directly investigated
Christ et al., 2007 [54]	BNP	CHF	ICD	VT/VF	Predictive of VT/VF	123	Prospective, single center	25 months	VT/VF	SCD not directly investigated
Verma et al., 2006 [56]	BNP, CRP	CHF	ICD	Appropriate ICD therapy	BNP predictive of appropriate ICD shocks	345	Prospective cohort single center	13 months	Appropriate ICD shocks	Independent predictor of SCD *
Vrotovec et al., 2013 [57]	BNP	CHF	None	SCD	Not predictive of SCD	512	Prospective single center	1 year	SCD	No effect
Bayes-Genis et al., 2007 [60]	NT-proBNP	CHF	None	SCD	Predictive of SCD	494	Prospective, multicenter (MUSIC study)	36 months	SCD	Independent predictor of SCD
Simon et al., 2008 [62]	NT-proBNP	DCM	None	nsVTs	Correlation with occurrence of nsVTs	30	Prospective, single center	21.6 ± 1.2 months	nsVTs	SCD not directly investigated
Scott et al., 2011 [63]	NT-proBNP, sST2, CRP, IL-6	CHF	ICD	Appropriate ICD therapy	NT-proBNP predictive of appropriate ICD therapy	156	Prospective, single center	15 ± 3 months	Appropriate ICD therapy	Factor associated with SCD *
Klingenberg et al., 2006 [64]	NT-proBNP	ICM	ICD	VA	Independent predictor of ICD therapy	50	Prospective, single center	1 year	Appropriate ICD therapy	Independent predictor for SCD *
Manios et al., 2005 [65]	NT-proBNP	ICM	ICD	VA	Predictive of VA	35	Prospective, single center	1 year	VA	SCD not directly investigated
Yu et al., 2007 [66]	NT-proBNP	ICM	ICD	VT/VF	Predictive of VT/VF	99	Prospective, single center	18 months	VT/VF	SCD not directly investigated
Levine et al., 2014 [67]	NT-proBNP, BNP	CHF	ICD	VA	Independently predictive of appropriate ICD therapy	695	Retrospective, multicenter	-	Appropriate ICD therapy	Independent predictor of SCD *

BNP, B-type natriuretic peptide; CAD, coronary artery disease; CHF, chronic heart failure; CMP, cardiomyopathy; CRP, C-reactive protein; CRT-D, cardiac resynchronization therapy – defibrillator; DCM, dilated cardiomyopathy; hsCRP, high sensitive C-reactive protein; ICD, implantable cardiac defibrillator; ICM, ischemic heart disease; IL-6, interleukin 6; nsVT, non sustained ventricular tachycardia; NT-proBNP, N-terminal pro-B-type natriuretic peptide; SCD, sudden cardiac death; sST2, soluble tool-like receptor-2; sVT, sustained ventricular tachycardia; TNFα, tumor necrosis factor alpha; VA, ventricular arrhythmias; VF, ventricular fibrillation; VT, ventricular tachycardia; sVT, sustained ventricular tachycardia; *If patient had ICD, appropriate therapy was defined as sudden cardiac death.

**Table 2:** Predictive value of “classic” biomarkers in hereditary cardiomyopathies.

Genetic	Biomarker	Underlying Condition	Pacemaker/ICD	Arrhythmias	Outcome	Number of Patients	Study Design	FU-Duration	Specific Endpoint	Effect on SCD
Bonny et al., 2010 [29]	CRP	ARVD/C	None	VT	Associated with VT	91	Prospective, single center	-	VT	SCD not directly investigated
Minami et al., 2018 [59]	BNP	HCM	None	SCD	Independent predictor of SCD	346	Prospective, single center	8.4 years	SCD	Independent predictor of SCD
Coats et al., 2013 [73]	NT-proBNP	HCM	None	SCD	Independent predictor of all-cause mortality but not of SCD	847	Prospective, single center	3.5 years	All-cause mortality (SCD)	No effect

ARVD, arrhythmogenic right-ventricular dysplasia/cardiomyopathy; BNP, N-type natriuretic peptide; CRP, C-reactive protein; HCM, hypertrophic cardiomyopathy; NT-proBNP, N-terminal pro-B-type natriuretic peptide; SCD, sudden cardiac death; VT, ventricular tachycardia.

**Table 3:** Predictive value of “classic” biomarkers in the general population.

General Population	Biomarker	Underlying Condition	Arrhythmias	Outcome	Number of Patients	Study Design	FU-Duration	Specific Endpoint	Effect on SCD
Hussein et al., 2013 [24]	CRP, IL-6	Adults aged 65 years or older	SCD	CRP and IL-6 are associated with SCD	5888	Subgroup analysis of prospective multicenter (Cardiovascular Health Study)	17 years (median 13.1 years)	SCD	Factor associated with SCD
Albert et al., 2002 [21]	hsCRP	Healthy men	SCD	Associated with SCD	97	Sub-study, prospective (Physician`s Healthy Study)	17 years	SCD	Factor associated with SCD
Korngold et al., 2009 [77]	NT-proBNP, hsCRP	Healthy women	SCD	Associated with SCD	32 828	Prospective, nested, case-control study	16 years	SCD	Factor associated with SCD
Hussein et al., 2013 [81]	hs-TnT	Ambulatory participants	SCD	Associated with SCD	4 431	Subgroup analysis of prospective multicenter (Cardiovascular Health Study)	13.1 years	SCD	Factor associated with SCD

AMI, acute myocardial infarction; CRP, C-reactive protein; hsCRP, high-sensitive C-reactive protein; IL-6, Interleukin 6; NT-proBNP, N-terminal pro-B-type natriuretic peptide; SCD, sudden cardiac death; VF, ventricular fibrillation; VT, ventricular tachycardia.

**Table 4:** Predictive value of novel or alternative biomarkers in heart failure.

Heart Failure.	Biomarker	Underlying Condition	Pacemaker/ICD	Arrhythmias	Outcome	Number of Patients	Study Design	FU-Duration	Specific Endpoint	Effect on SCD
Daidoji et al., 2012 [85]	H-FABP	CMP	ICD	Appropriate ICD shocks or cardiac death	Correlation with levels of H-FABP	107	Prospective, single center	33.6 month	appropriate ICD shock or cardiac death	Independent predictor of SCD *
Nodera et al., 2018 [86]	Uric Acid	CHF	ICD	VT	Uric Acid predicts VT	56	Prospective, single center	30 ± 8 months	appropriate ICD shock	Independent predictor of SCD *
Flevari et al., 2012 [87]	MMP-9	CHF	ICD	VT	MMP-9 and PICP are predictive of VT	74	Prospective, single center	1 year	appropriate intervention for sVT	Independent predictor of SCD *
Sardu et al., 2018 [28]	sST2, NT-proBNP, CRP	HF patients with metabolic syndrome	ICD	Appropriate ICD therapy	Prediction of ICD shocks	MS: 99 vs. Non-MS: 107	Prospective, multicenter	1 year	appropriate and inappropriate ICD therapy	Independent predictor of SCD *
Francia et al., 2014 [88]	OPN, galectin-3	CHF	ICD	VF, VT	OPN and galectin-3 predict sVT/VF	75	Prospective, single center	29 ± 17 months	first sVT/VF	Independent predictor of SCD *
Ahmad et al., 2014 [89]	NT-proBNP, sST2, galectin-3	CHF	None	SCD	Positive with NT-proBNP, mildly incremental when combined with novel biomarkers	813	Sub-study, Prospective, multicenter (HF-ACTION)	2.5 years	SCD	Independent predictor of SCD
Skali H et al., 2016 [90]	sST2	HF	CRT Registry	VT	Predictive of VT	684	Sub-study, prospectively, multicenter (MADIT)	1 year	VT /VF or death	SCD not directly investigated
Pascual-Figal et al., 2009 [91]	sST2 NT-proBNP	CHF	None	SCD	Positive when Combined with NT-proBNP levels	36 SCD matched 63 Controls	Sub-group analysis, case-control design of prospective, multicenter MUSIC study	3-years	SCD	Independent predictor of SCD

CMP, cardiomyopathy; CHF, chronic heart failure; CRP, C-reactive protein; CRT, cardiac resynchronization therapy; CVD, cardiovascular death; H-FABP, Heart-type fatty acid binding protein; ICD, implantable cardiac defibrillator; MMP-9, matrix metallo-proteinase; MS, metabolic syndrome; NTproBNP, N-terminal pro-B-type natriuretic peptide; OPN, Osteopontin; PICP, procollagen type I carboxyterminal peptide; SCD, sudden cardiac death; sST2, soluble toll-like receptor-2; sVT, sustained ventricular tachycardia; VT, ventricular tachyarrythmia; VF, ventricular fibrillation;. *If patient had ICD, appropriate ICD therapy was defined as sudden cardiac death.

**Table 5:** Predictive value of novel or alternative biomarkers in hereditary cardiomyopathies.

Genetic	Biomarker	Underlying Condition	Pacemaker/ICD	Arrhythmias	Outcome	Number of Patients	Study Design	FU-Duration	Specific Endpoint	Effect on SCD
Oz et al., 2017 [92]	Galectin-3	ARVD	ICD	VF, VT	Correlation with Galectin-3	29 vs. 24 controls	Retrospective, multicenter	-	nsVT/sVT	SCD not directly investigated
Daidoji et al., 2016 [93]	H-FABP	Brugada syndrome	ICD	Appropriate ICD shock, VF	Correlation with VA	31	Prospective, single-center	5 years	appropriate ICD shock	Independent predictor of SCD *
Zachariah et al., 2012 [94]	MMP3	HCM	ICD	VT/VF	MMP3 predicts VA	45	Retrospective, single Center	6 months	CA, sVT/VF with ICD shock	SCD not directly investigated
Emet et al., 2018 [95]	Galectin-3	HCM	ICD	SCD	Predictive 5 year risk of SCD	52	Cross-sectional data	-	Correlation between the estimated 5-year risk of SCD	SCD not directly investigated

ARVD, arrhythmogenic right ventricular dysplasia; CA, cardiac arrest; HCM, hypertrophic cardiomyopathy; H-FABP, Heart-type fatty acid binding protein; ICD, implantable cardiac defibrillator; MMP-9, matrix metallo-proteinase; nsVT, non-sustained ventricular tachycardia; SCD, sudden cardiac death; VA, ventricular arrhythmias; VF, ventricular fibrillation; VT ventricular tachycardia. * If patient had ICD, appropriate ICD therapy was defined as sudden cardiac death.

**Table 6:** Predictive value of novel or alternative biomarkers in the general population.

General Population	Biomarker	Underlying Condition	Arrhythmias	Outcome	Number of Patients	Study Design	FU-Duration	Specific Endpoint	Effect on SCD
Kunutsor et al., 2016 [96]	Fibrinogen	Non	SCD	Fibrinogen is associated with SCD	1773	Prospective cohort study, multicenter	22 years	SCD	Independent predictor of SCD
Yamade at al., 2011 [97]	Uric acid	Non-specific LVH	VT	Uric acid predicts VT	167	Prospective, single center	24 h	Correlation with VT in 24h- Holter ECG	SCD not directly investigated
Deo et al., 2010 [98]	Cystatin C	Age/no cardio-vascular disease	SCD	Correlation with cystatin C	4465	Subgroup analysis of Prospective, multicenter CHS (Cardiovascular health study)	11.2 years	SCD	Independent predictor of SCD
Jouven et al., 2001 [99]	circulating nonesterified fatty acids	Non	SCD	independent risk factor for SCD	5250	Cohort-Study(Paris Prospective Study I)	22 years	SCD	Independent predictor of SCD

ECG, electrocardiogram; LVH, left ventricular hypertrophy; SCD, sudden cardiac death; VT, ventricular tachycardia.
